# The enigma of small heat shock protein phosphorylation

**DOI:** 10.3389/fphar.2024.1486245

**Published:** 2024-10-23

**Authors:** Matthias Gaestel

**Affiliations:** Hannover Medical School, Institute of Cell Biochemistry, Hannover, Germany

**Keywords:** MAPKAP kinases, sHSPs, phosphorylation, chaperone, inflammation

## Introduction

In a recent Forum contribution to Trends in Pharmacological Sciences, (Wang and Pratt, 2024) discussed the potential of targeting post-translational modifications (PTMs) of small heat shock proteins (sHsps), such as phosphorylation, glycosylation and glycation (Wang and Pratt, 2024). They conclude that simple loss-of-modification studies, using mutations at the modification sites, can also cause indirect structural effects. Accordingly, they emphasized that identifying the enzymes responsible for specific modifications is crucial for developing inhibitors against particular sHsp modifications. However, additional aspects should be considered in this approach, as demonstrated here in particular for sHsp phosphorylation.

## Discussion and prospective

**FIGURE 1:**
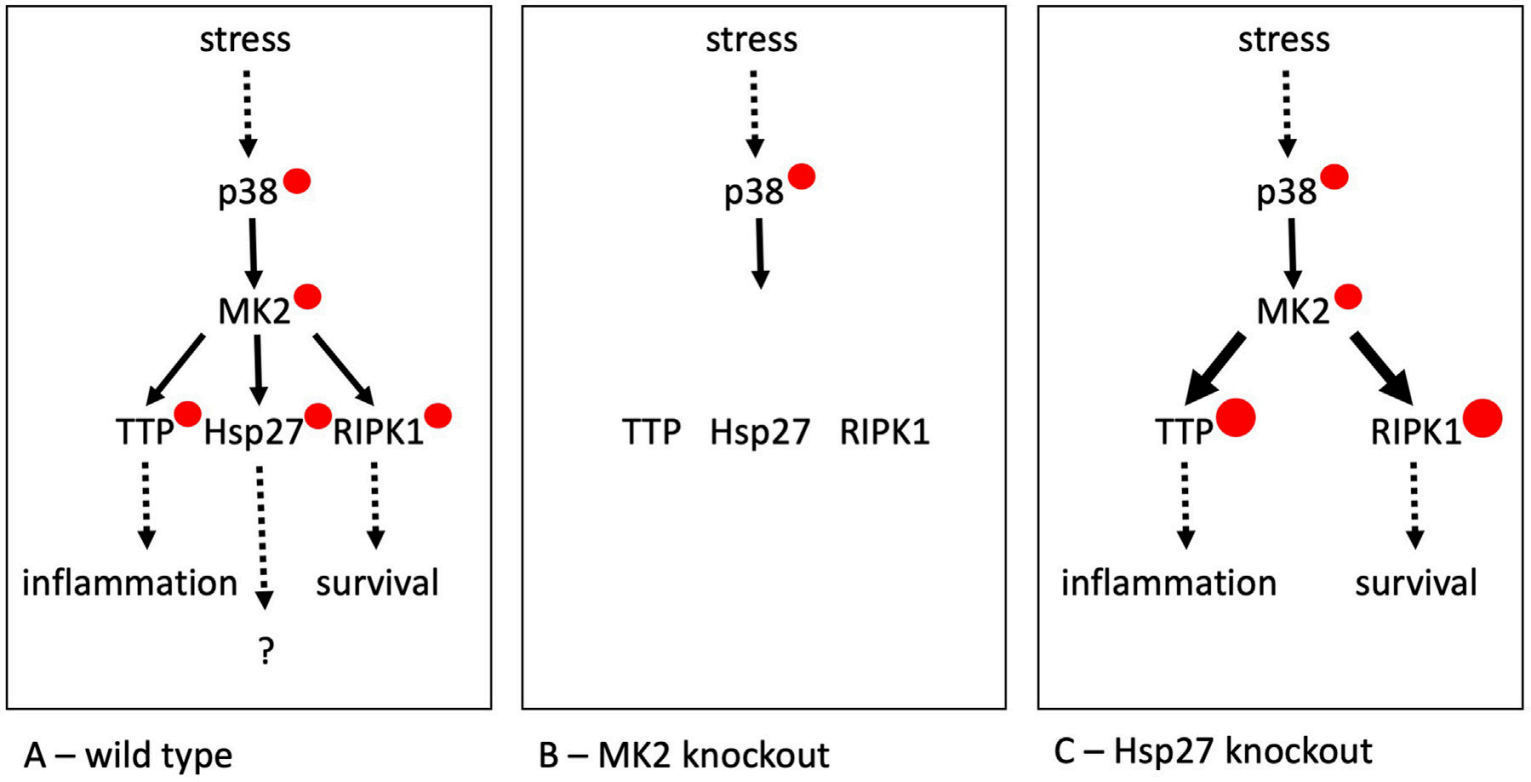
The stress-dependent p38/MK2 pathway and its substrates in wild type **(A)**, MK2-deficient **(B)** and Hsp27-deficient cells **(C)**. p38 – p38 MAPK, MK2 – MAPKAP kinase 2, TTP – tristetraprolin, RIPK1 – TNF-receptor-interacting protein kinase 1, Hsp27 – Hspb1. Solid arrows represent phosphorylation events, with red circles indicating the resulting phosphorylations. For the MK2 substrates, the size of the red circles should reflect the relative level of phosphorylation. The number of distinct phosphorylation sites on the protein kinases and substrates are not depicted in this figure.

Hsp27 (HSPB1) is the most extensively studied mammalian sHsp, with its major PTMs – phosphorylations at two serine residues conserved in mice and humans – known for over 30 years (Gaestel et al., 1991). Only 1 year after these PTM sites were identified, the primary enzyme responsible was characterized as MAPK-activated protein kinase 2 (MK2), which is itself phosphorylated and activated by the stress-dependent protein kinase p38 MAPK (Stokoe et al., 1992; Rouse et al., 1994) (Figure 1). First, this signaling pathway elegantly explained the stress-dependent phosphorylation of Hsp27. Second, the genetic deletion of MK2 in mice revealed an unexpected yet essential role of this enzyme in p38 MAPK-dependent signaling for the biosynthesis of inflammatory cytokines (Kotlyarov et al., 1999).

Since p38 MAPK inhibitors failed in the process to develop effective novel anti-inflammatory therapies, many pharmaceutical companies began screening for inhibitors targeting MK2. Nowadays, ATP-competitive (PF3604422), covalent (CC99677) and activation-preventing (ATI-450) inhibitors against MK2 are available, all of which effectively inhibit Hsp27 phosphorylation (Wang et al., 2018; Ronkina and Gaestel, 2022).

Mechanistically, it was found that MK2 stabilizes cytokine mRNAs through direct phosphorylation of the AU-rich RNA element-binding protein tristetraprolin (TTP) (Tiedje et al., 2016) and supports the survival of immune and other cells by directly phosphorylating the central cell-death kinase RIPK1 (Oberst, 2017). As a result, there are at least three physiological relevant substrates of MK2: Hsp27, TTP and RIPK1 (Figure 1). This number is likely underestimated, as more MK2 substrates have been identified (Ronkina and Gaestel, 2022). Considering that the approximately 520 human protein kinases phosphorylate over 100,000 non-redundant sites in the proteome, each kinase, on average, targets around 200 sites.

While TTP is expressed in many but not all cell types, RIPK1 is ubiquitously expressed. Inhibition of Hsp27 phosphorylation by the highly selective MK2 inhibitors will simultaneously inhibit RIPK1 phosphorylation and, where present, TTP phosphorylation. This makes it impossible to differentiate between the effects of phosphorylation of Hsp27, RIPK1 and TTP and other MK2 substrates. Therefore, it is not possible to identify the function of these particular PTMs of Hsp27 by inhibiting the responsible enzyme.

Wang and Pratt (2024) also described our lab’s use of mutants to analyze the phosphorylation-dependent function of mouse Hsp27 in transiently transfected cells. Our results demonstrated that the oligomers of ectopically expressed Hsp27 with phospho-mimicking mutations decrease in size, correlating with reduced chaperone and stress-protective properties. Additionally, we demonstrated that *in vitro* phosphorylation of recombinant Hsp27 similarly results in a reduction in oligomeric size (Rogalla et al., 1999). It is worth noting that we subsequently analyzed endogenous Hsp27 in MK2-deficient cells. In the absence of MK2, the stress-dependent disaggregation of Hsp27 complexes was impaired, and Hsp27 showed delayed subcellular accumulation in stress granules (Vertii et al., 2006). However, in this analysis, we could not exclude the possibility that the observed cellular effects were at least partly due to changes in TTP-dependent cytokine production or RIPK1’s apoptotic activity in the MK2-deficient cells.

Two different mouse knockout lines of Hsp27 have been generated. The first uses lacZ replacement of a large part of Hsp27 and reports no significant phenotype resulting from the deletion (Huang et al., 2007). Interestingly, in the second approach using a loxP-mediated complete deletion of Hsp27 in mice, the authors detected a small but significant increase in cytokine production (Crowe et al., 2013). It is possible that the deletion of Hsp27 leads to “over-phosphorylation” of the other substrates of MK2 involved in inflammation stimulation such as TTP and RIPK1, thereby masking the direct function of Hsp27 (Figure 1).

The only approach not yet undertaken to understand Hsp27 phosphorylation is a knock-in strategy in mice, utilizing CRISPR-directed mutagenesis to alter the phosphorylation sites (through deletion and/or phospho-mimicking replacements). However, given that the phenotypes observed in the complete Hsp27 knockout mice are relatively mild or even absent, it is questionable whether significant effects will be seen with this knock-in strategy. Nevertheless, if phosphorylation has a substantial role in Hsp27 function, a phospho-mimic knock-in could still yield a functionally relevant phenotype. The potential redundancy among the ten different existing human sHsps and their ability to form heterogeneous complexes could further complicate the interpretation of the results. Nonetheless, the author remains optimistic that new ideas and approaches will eventually elucidate the phosphorylation-dependent *in vivo* function(s) of Hsp27.

## References

[B1] Crowe J. Aubareda A. McNamee K. Przybycien P. M. Lu X. Williams R. O. (2013). Heat shock protein b1-deficient mice display impaired wound healing. PLoS One 8, e77383. 10.1371/journal.pone.0077383 24143227 PMC3797036

[B2] Gaestel M. Schröder W. Benndorf R. Lippmann C. Buchner K. Hucho F. (1991). Identification of the phosphorylation sites of the murine small heat shock protein hsp25. J. Biol. Chem. 266, 14721–14724. 10.1016/s0021-9258(18)98746-6 1860870

[B3] Huang L. Min J.-N. Masters S. Mivechi N. F. Moskophidis D. (2007). Insights into function and regulation of small heat shock protein 25 (HSPB1) in a mouse model with targeted gene disruption. Genes. N. Y. N.Y. 45 (45), 487–501. 10.1002/dvg.20319 17661394

[B4] Kotlyarov A. Neininger A. Schubert C. Eckert R. Birchmeier C. Volk H. D. (1999). MAPKAP kinase 2 is essential for LPS-induced TNF-alpha biosynthesis. Nat. Cell. Biol. 1, 94–97. 10.1038/10061 10559880

[B5] Oberst A. (2017). MK2 balances inflammation and cell death. Nat. Cell. Biol. 19, 1150–1152. 10.1038/ncb3619 28960200

[B6] Rogalla T. Ehrnsperger M. Preville X. Kotlyarov A. Lutsch G. Ducasse C. (1999). Regulation of Hsp27 oligomerization, chaperone function, and protective activity against oxidative stress/tumor necrosis factor alpha by phosphorylation. J. Biol. Chem. 274, 18947–18956. 10.1074/jbc.274.27.18947 10383393

[B7] Ronkina N. Gaestel M. (2022). MAPK-activated protein kinases: servant or partner? Annu. Rev. Biochem. 91, 505–540. 10.1146/annurev-biochem-081720-114505 35303787

[B8] Rouse J. Cohen P. Trigon S. Morange M. Alonso-Llamazares A. Zamanillo D. (1994). A novel kinase cascade triggered by stress and heat shock that stimulates MAPKAP kinase-2 and phosphorylation of the small heat shock proteins. Cell. 78, 1027–1037. 10.1016/0092-8674(94)90277-1 7923353

[B9] Stokoe D. Engel K. Campbell D. G. Cohen P. Gaestel M. (1992). Identification of MAPKAP kinase 2 as a major enzyme responsible for the phosphorylation of the small mammalian heat shock proteins. Febs Lett. 313, 307–313. 10.1016/0014-5793(92)81216-9 1332886

[B10] Tiedje C. Diaz-Muñoz M. D. Trulley P. Ahlfors H. Laass K. Blackshear P. J. (2016). The RNA-binding protein TTP is a global post-transcriptional regulator of feedback control in inflammation. Nucleic acids Res. 44, 7418–7440. 10.1093/nar/gkw474 27220464 PMC5009735

[B11] Vertii A. Hakim C. Kotlyarov A. Gaestel M. (2006). Analysis of properties of small heat shock protein Hsp25 in MAPK-activated protein kinase 2 (MK2)-deficient cells MK2-DEPENDENT insolubilization of Hsp25 oligomers correlates with susceptibility to stress. J. Biol. Chem. 281, 26966–26975. 10.1074/jbc.m602134200 16840785

[B12] Wang B. Pratt M. R. (2024). Potential for targeting small heat shock protein modifications. Trends Pharmacol. Sci. 45, 583–585. 10.1016/j.tips.2024.04.002 38704305 PMC11227382

[B13] Wang C. Hockerman S. Jacobsen E. J. Alippe Y. Selness S. R. Hope H. R. (2018). Selective inhibition of the p38α MAPK–MK2 axis inhibits inflammatory cues including inflammasome priming signals. J. Exp. Med. 215, 1315–1325. 10.1084/jem.20172063 29549113 PMC5940269

